# Zoonotic diseases risk perception and infection prevention and control practices among poultry farmers in the Buea Health District, Cameroon: A one health perspective

**DOI:** 10.14202/vetworld.2022.2744-2753

**Published:** 2022-11-30

**Authors:** Marie Ebob Agbortabot Bissong, Johnny Castro Nganjo Lyombe, Emmanuel Asongalem, Robert Bongji Ngamsha, Nicholas Tendongfor

**Affiliations:** 1 Department of Biomedical Sciences, University of Bamenda, Bambili, Cameroon; 2 Department of Microbiology and Parasitology, University of Buea, Buea, Cameroon; 3 Department of Public Health and Hygiene, University of Buea, Buea, Cameroon; 4 Department of Biomedical Sciences, University of Buea, Buea, Cameroon

**Keywords:** Cameroon, control practices, knowledge, poultry farmers, risk perception, Zoonotic diseases

## Abstract

**Background and Aim:**

Livestock are associated with pathogenic microbes and farm workers play a significant role in the transmission of zoonotic diseases (ZDs). Lack of awareness of exposure risk among farmers may influence their farm practices, thereby enhancing the spread of diseases on farms and to the community. This study was aimed at evaluating the knowledge, risk perception, and prevention and control practices of ZDs among poultry farmers to provide baseline data for establishing a “One Health” practical approach to reducing ZD transmission in poultry farms.

**Materials and Methods:**

Using the exponential discriminative snowball technique, a community-based cross-sectional study involving poultry farmers was carried out in the Buea Health District from April to July 2021. Six feed-producing mills were used as focal points to identify and recruit farmers who were also referred to other farmers. Questionnaires were used to collect data related to participants’ knowledge, risk perception, and prevention and control practices of ZDs. Descriptive analyses were performed for all variables while the chi-square test and logistic regression analysis were used to determine associations at 95% confidence level.

**Results:**

In all, 183 poultry farms and 207 workers were enrolled in the study. Despite being aware that animal diseases can be transmitted to humans, most participants showed poor knowledge (54.6%), low-risk perception (51.7%), and poor prevention/control practices (54.1%) on ZDs. The majority did not consider coming in contact with birds’ body fluid (blood) or apparently healthy birds to be a risk of infection. More participants with small farms (<500 birds) had low-risk perception of ZDs than those with larger farms (>1000 birds) (p = 0.03). Furthermore, most participants reported practicing hand washing but they neither used protective devices such as gloves and face masks, and >50% would not invite veterinary professionals to their farms. There was a significant association between risk perception and knowledge (p = 0.007; CI = 1.257–4.200) as well as between risk perception and prevention/control practice (p = 0.002; CI = 1.451–4.867).

**Conclusion:**

Poultry farm workers in Buea had poor knowledge and perception of ZD risk and this might have contributed to their poor prevention/control practices on the farms. Enhanced informal education of poultry farmers through training workshops and seminars will improve their knowledge and skills on ZD transmission risk and prevention.

## Introduction

Zoonotic diseases (ZDs) or zoonoses are diseases that are transmitted from animals to humans [1]. The close proximity between humans and animals as pets, livestock rearing, and game-hunting may increase the possibility of transmission of ZDs to humans. It is estimated that more than 6 out of 10 infectious diseases originate from animals and 3 out of 4 new or emergent infectious diseases in humans come from animals [2]. Most animals serve as reservoirs for the emergence or re-emergence of pathogenic microbes. Most of these microbes dwell in the gastrointestinal tract of these animals as commensals but may become pathogenic when transmitted to a suitable human host. Zoonotic diseases are among the most recurrent and feared risks to humans. The emergence and re-emergence of ZDs and the devastating impact on human health are a growing concern around the globe [3]. Globally, 2.5 billion cases are estimated to be related to ZDs yearly, resulting in 2.7 million deaths [4]. Zoonotic diseases account for about 25% of the infectious disease burden in low-income countries [4]. A combined disease burden is observed in individuals in tropical and subtropical Africa, where there is a likelihood of ZDs coinfection with other infectious diseases such as malaria, tuberculosis and HIV. These associated factors may increase the severity of diseases and the susceptibility of individuals to zoonotic agents, thus enhancing their spread at the community level [5]. Central Africa has been identified as a hotspot for emerging infectious diseases and ZDs due to factors such as population growth, urbanization, political and social disruption, agriculture and livestock intensification, deforestation, and climate change [6]. Zoonotic diseases are caused by a range of pathogens such as viruses, bacteria, fungi, protozoa and helminths. Some ZDs associated with birds include avian influenza, Newcastle disease, avian tuberculosis, erysipelas, ornithosis, cryptococcosis, histoplasmosis, salmonellosis, cryptosporidiosis, campylobacteriosis, and escherichiosis (colibacillosis) [7, 8]. About 70% of emerging viral diseases represent zoonoses, with such prominent examples being HIV/AIDS, influenza, West Nile virus encephalitis, SARS, Ebola virus disease, Marburg virus disease, hantavirus pulmonary syndrome, and the most recent SARS-CoV-2 (causative agent of the on-going COVID-19 pandemic) [9, 10]. The transmission of ZDs can be through direct contact, indirect contact, vector-borne, foodborne, and water borne [3, 11]. Foodborne ZDs may be transmitted across the food chain; notably, during slaughtering, meat processing, and handling of food products of animal origin [4, 7]. Zoonotic diseases prevention methods differ for each pathogen. However, several practices have been shown to be effective in reducing risk at the community and personal levels [11, 12]. Safe and appropriate guidelines for animal care in the agricultural sector help to reduce the potential for foodborne ZD outbreaks through foods such as meat, eggs, dairy, or even some vegetables [11–13]. Standards for clean drinking water and waste removal, as well as protection of surface water in the natural environment are also important and effective [14, 15]. Education campaigns to promote hand washing after contact with animals and other behavioral adjustments can reduce community spread of ZDs [14, 15]. The livestock population of Cameroon is estimated at more than 90 million and includes 72 million poultry, 9 million small ruminants, 5 million cattle, and 3 million swine [16]. It is estimated that over 70% of the population is engaged in small-scale livestock agriculture with the majority being poultry farmers [16]. The poultry industry in Cameroon has experienced substantial growth, perhaps due to the ban on the importation of frozen chicken [17]. This has led to the creation of many poultry farms managed by unskilled workers. In such a resource-scarce setup where access to health and veterinary services are limited, inappropriate farm practices may be associated with increased likelihood of exposure to zoonotic pathogens and outbreaks of ZDs. A recent study reported high biosecurity risk in some poultry farms in the Centre, Littoral and West regions of Cameroon and attested to the fact that there was a significant relationship between biosecurity levels and disease outbreaks on the farms [18]. Despite these risks associated with poultry farms in the Country, there is a paucity of information on ZDs, especially in the South West region.

Considering that this study was the first to describe risk perception of ZDs among poultry farmers in Cameroon, it will create awareness of the occurrence and risk of ZDs transmission among poultry farmers in the study area. The study will also add to the literature the types of diseases common in poultry farms in the study area. Identifying the prevalent diseases, the level of awareness and prevention measures of ZDs among farmers in a semi-urban resource-limited community will provide baseline information for establishing “One health” practical approaches to reduce ZD transmission in poultry farms and in the community.

This study aimed to evaluate the knowledge, risk perception and prevention/control practices of ZDs among poultry farmers in the Buea Health District (BHD) and to elucidate the relationship between these factors.

## Materials and Methods

### Ethical approval

Ethical approval for this study was obtained from the Institutional Review Board of the Faculty of Health Sciences of the University of Buea (IRB00008917-US Office for Human Research Protection IORG007426) protocol number 2021/1446-04/UB/SG/IRB/FHS.

### Study period and location

**Figure-1:**
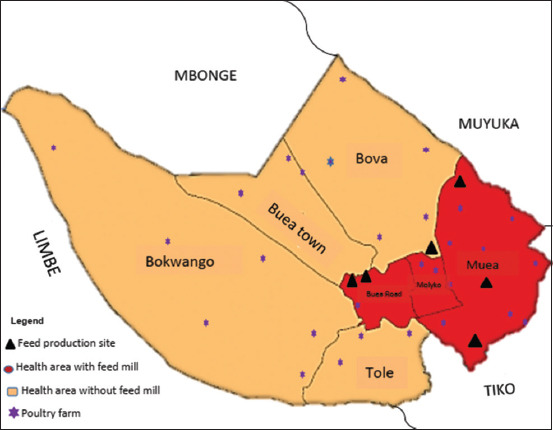
Map of Buea Health District showing the study sites [Source: shorturl.at/pDGH4].

The study was conducted from April to July 2021. The study was a descriptive cross-sectional quantitative study. This study was conducted in the BHD which is made up of seven health areas (HAs) namely: Bokwoango, Bova, Buea road, Buea town, Molyko, Muea, and Tole (Figure-1). These areas represent the rural and semi-urban communities of the city of Buea. Buea is a cosmopolitan city consisting of about 67 villages with a surface area of 870 sq/km [19]. Buea is bound to the north by the tropical forest on the slope of Mount Cameroon, which extends to the Atlantic Ocean. The town also shares boundaries with other major towns like the Limbe to the Southwest, Tiko to the Southeast, Muyuka municipality to the east, and Idenau district to the west. The dominant economic activity is agriculture, which forms the backbone of the local economy. The inhabitants are engaged in both crop cultivation and livestock rearing, including animals such as chicken, pigs, cows, goats, and rabbits.

### Target population

The target population was made up of all poultry farmers, including farm owners (employers) and workers (employees) in the study area who carried out daily farm procedures within the study period. Participants who consented and who met the inclusion criteria (farm size of at least 100 birds, residence at the BHD and easy accessibility to the individual’s house or farm) were included in the study.

### Sample size calculation

The sample size for this study was determined using the formula [20]:


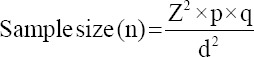


Where Z = 1.96, confidence level 95%, p = 0.25, the proportion obtained from a previous study [21], q = 1–p, e = 5% or 0.05 which is the level of precision.


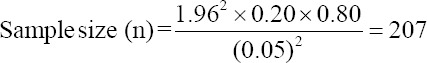


Therefore, the estimated minimum sample size for this study was 207.

### Sampling technique

The snowball technique; notably, the exponential discriminative technique, was used to recruit participants. Six main feed production sites in Muea, Great Soppo, Bonduma, Bwitingi, and Mile 16 (Figure-1) were identified and all poultry farmers who came to these shops to purchase feed were enrolled in the study. These participants were referred to other poultry farmers who were equally enrolled. The referral was continued until the sample size was met. Participants who did not meet the expected criteria were rejected.

### Data collection procedure

Data were collected using open-ended and closed-ended questionnaires by well-trained personnel. The questionnaires were structured to reflect the following objectives:

#### Socio-demographic and farm characteristics

This section comprised 11 questions reflecting the sociodemographic characteristics of the participants and various farm attributes. The farm characteristics evaluated were farm size, duration of being a poultry farmer, current size of flock, yearly flock capacity of farm, average number of birds per year, types of birds on the farm, mixed farming, and position of participant on the farm.

#### Knowledge of ZDs

To evaluate participants’ knowledge on ZDs, seven questions related to type, symptoms and transmission of ZDs were assessed. Each question had a score of “1” for a correct response and “0” for a wrong response. A knowledge score was developed as previously reported [22, 23] based on participants’ responses to the 7 questions. The maximum score for knowledge was 7 and the mean score was 4.87 ± 1.56. Scores above the mean (>4.87) were categorized as “good knowledge” while mean scores and below the mean (≤4.87) were classified as “poor knowledge.”

#### Risk perception of ZDs among poultry farmers

A group of eight questions were used to determine participants’ risk perception of ZDs. These questions were based on the participant’s ability to identify their likelihood of exposure to ZDs, their concern for them or their colleagues, clients or family members contracting a ZD and their awareness of biosecurity guidelines in poultry farming. A risk perception score was developed based on participants’ responses as previously reported [22, 23]. The responses were categorized as follows: ability to identify their likelihood of exposure to ZDs (very likely = 3, likely = 2, A little likely = 1, and Not likely = 0) from various procedures, their level of concerned (“very concern” scored 3, “concerned” scored 2, “a little concerned” scored 1, and “not concerned” scored 0) for them or colleague or clients or family members contracting a ZD and their awareness of biosecurity guidelines in poultry farming (“Yes” scored 1 and “No” scored 0). The maximum score was 15 and the mean score was (6.58 ± 4.39). Scores ≤6.58 were considered “low-risk perception” while those >6.58 were considered “high-risk perception.”

#### Infection prevention and control practices in poultry farms

To assess participants’ prevention and control practices, 11 questions were asked. These included aspects of hand hygiene, use of personal protective equipment (PPE), actions taken when birds are either sick or dying in large numbers (disease outbreak), regularity of veterinary visits or control in their farms and actions taken on dead birds. Prevention and control practice score was developed based on participant’s responses to all the questions, as previously reported [22, 23]. The responses were categorized as follows: For frequency of hand hygiene practice and use of PPE (gloves, face masks, protective boots, and jackets), “Always” = 2, “sometimes” = 1, and “Never” = 0. For actions taken when birds were sick or died in their numbers, Veterinarian visit the responses were “Yes” = 1 or “No” = 0 and frequency of Vet visit (“Frequently or more frequently” = 1 and “less frequently” = 0); and “what do you do with the dead birds?” (“burn and burry” = 1 and “eat or sell or throw in the bin or backyard” = 0). The maximum score was 13 and the mean score was 6.21 ± 2.67. Prevention/control practice scores were categorized as “good” when scores were >6.21 or “poor” when scores were ≤6.21.

### Statistical analysis

Questionnaires were checked for proper completion of the collection from the participants. Questionnaires with >20% unanswered questions were rejected. Data were summarized in a Microsoft Excel 2016 spreadsheet, exported, and analyzed using SPSS version 25. Descriptive and analytic statistics were used to analyze variables with frequencies and proportions predominantly used to describe the data. Independent variables were created from the socio-demographic and farm characteristics, knowledge, risk perceptions, and prevention and control practices. To create outcome variables, a unique score card was used for the responses. Each study participant was assigned a score that reflected the stringency of his or her response. To measure responses to these independent factors, the scoring system ranged between the following: 0 and 7 points for knowledge, 0 and 15 for risk perception, and 1 and 13 for infection prevention and control. The score range was further categorized into “poor or low” (≤mean score) and “good or high” (>mean score) to keep them as binary variables. Associations between the outcome and independent variables were first subjected to univariate analyses using Chi-square tests. All factors found to be statistically significant were subsequently analyzed using multivariate logistic regression models to control for confounders and to test for effect modification. p < 0.05 was considered statistically significant.

## Results

### Socio-demographic data of poultry farmers and characteristics of farms

A total of 207 farm workers were enrolled in the study, the majority were males (72.9%) compared to females (27.1%). Most (36.2%) of the participants were within the age group of 26–35 years with a mean age of 35.5 ± 10.48 years. Most of the respondents (47.3%) had a university level of education and a few (1.0%) had no formal education. More than half (73.9%) of the respondents had been working as poultry farmers for <5 years. Meanwhile, majority of the participants were farm owners (82.1%) while the others were employees (14.5%) and business partners (3.4%).

Table-1 shows the characteristics of various farms. Most of the farms were located in Muea (27.1%), Bova (25.2%), and Buea Road (20.8%) HAs. The total number of birds on the farms varied from 100 to more than 8000 birds. In 67.6% of the farms only chickens were kept and out of this, 96.1% reared only broilers species which was meant for meat production. Some farms (32.4%) practiced mixed farming with the predominant animals being pigs (26.6%). Almost half of the respondents (49.3%) had small farms (<500 birds). The average number of birds that were currently present on the farms was 519.47 ± 773.185 birds, while the average yearly production of birds was 3040.69 ± 3994.015 birds.

**Table-1:** Characteristics of poultry farms in the study area.

Variables	Categories	Frequency	Proportion (%)
Farm size	Large (above 1000)	44	21.3
	Medium (500–1000)	61	29.5
	Small (below 500)	102	49.3
Type of birds reared	Broilers	199	96.1
	Layers	3	1.4
	Brahma	3	1.4
	Local fowls	1	0.5
	Mixed birds	1	0.5
Other animals in the farm	Pigs	55	26.6
	Goats	8	3.9
	Dog	2	1.0
	Others	2	1.0
	None	140	67.6
Location of farm	Bova	46	25.2
	Buea road	38	20.8
	Bokwango	10	5.5
	Molyko	16	8.7
	Muea	50	27.3
	Buea town	16	8.7
	Tole	7	3.8

### Knowledge of ZDs among poultry farmers

In general, 113/207 participants (54.6%) had poor knowledge about zoonotic poultry diseases as opposed to 45.4% with good knowledge. Although majority of the participants knew how to identify sick birds (99.5), knew that infections could be obtained from sick birds (60.4%), most of them (63.8%) did not know that infections could be acquired from apparently healthy birds (Table-2).

**Table-2:** Distribution of responses to knowledge attributes among poultry farmers.

Variables	Categories	Proportion of farmers (%)
Zoonotic disease definition	Yes	134 (64.7)
	No	73 (35.3)
Infection from apparently birds	Yes	75 (36.2)
	No	132 (63.8)
Infection from sick bird	Yes	125 (60.4)
	No	82 (39.6)
Identify sick birds	Yes	206 (99.5)
	No	1 (0.5)

### Common signs, symptoms, and poultry diseases reported by poultry farmers

**Figure-2:**
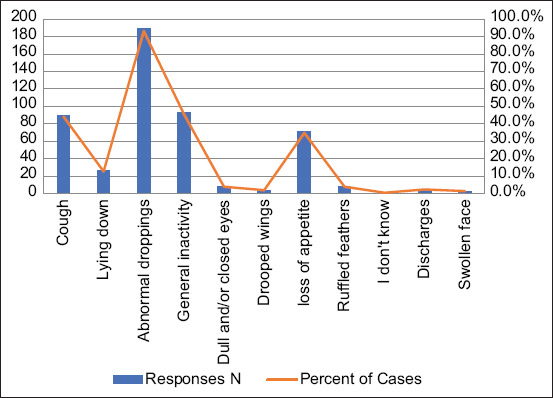
Common signs and symptoms of poultry diseases reported by the poultry farmers.

**Figure-3:**
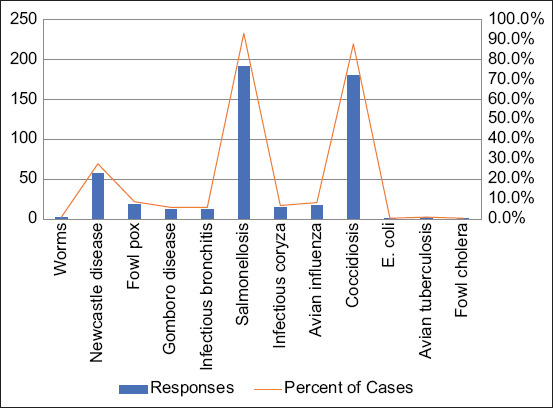
Poultry diseases common on farms in the study area. Worms refers to worm (helminthic infestation) while *E. coli* refers to *E. coli* infection (colibacillosis). *E. coli=Escherichia coli*.

The predominant signs and symptoms reported by participants included abnormal droppings (190/204, 93.1%), general inactivity (93/204, 45.6%), cough (90/204, 44.1%), and loss of appetite (71/204, 34.8%) (Figure-2). In total, 12 poultry diseases were identified by the farmers to be common in poultry farms among which were salmonellosis, commonly called “white diarrhea” (191/205, 93.2%), coccidiosis also known as “brown diarrhea” (180/205, 87.8%), and Newcastle disease (57/205, 27.8%) (Figure-3) Of the 12 diseases reported by the farmers, four were ZDs; namely, salmonellosis, Newcastle disease, avian influenza, avian tuberculosis, and colibacillosis (*Escherichia coli* infections).

**Figure-4:**
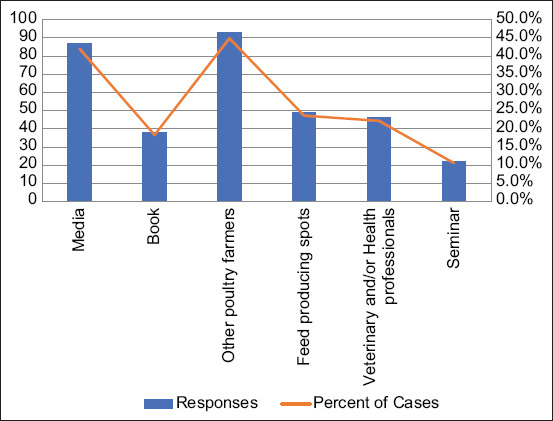
Sources of information about Poultry zoonotic diseases reported by poultry farmers.

In this study, poultry farmers received information about poultry and ZDs from different sources, mainly from other poultry farmers (44.9%) and media (TV, radio, and internet) (42%) (Figure-4).

A bivariate analysis between demographic variables and knowledge of ZDs revealed significant differences between knowledge and age (χ^2^ = 13.45; p = 0.009); level of education (χ^2^ = 19.76; p < 0.001); and position in the farm (χ^2^ = 8.14; p = 0.017). In general, participants between the ages 26 and 55 years had better knowledge of ZDs than those <26 years and those >55 years. Meanwhile, participants with a higher level of education (University) were more knowledgeable than the other groups. Furthermore, 50% of the poultry farmers had good knowledge than employees (23.3%) (χ^2^ = 8.14; p = 0.017).

### Risk perception of ZDs among poultry farmers

A total of 51.7% of participants had a low level of perception while 48.3% had high risk perception of ZDs. Participants were asked to state their likelihood of exposure to ZDs when in contact with bird’s body fluid (blood), carcass, faces, healthy, and sick birds. Most participants did not consider coming in contact with bird’s body fluid (blood) (68.1%) or apparently healthy birds (76.8%) to be a risk of infection. When asked “How concerned are you that you, your colleague, clients or family members could contract a ZD?” Most participants were less concerned or did not see any risk of them or others contracting a ZD. In addition, majority (82.1%) of participants were not aware of biosecurity guidelines in poultry farming.

There was an association between participants’ risk perception and demographics; notably, level of education, position in farm and farm size (p < 0.05) (Table-3). In general, participants who were farm employees had high risk perception compared to farm owners and business partners while those with university education had higher risk perception than participants with lower levels of education. Furthermore, more participants with small farms (<500 birds) had low risk perception of ZDs than those with large farms (>1000 birds).

**Table-3:** Association between participants’ risk perception and demographic characteristics.

Variables	Categories	Level of perception		p- value

		Low (%)	High (%)	
Age group	15–25	13 (37.1%)	22 (62.9%)	0.314
	26–35	39 (52.0%)	36 (48.0)	
	36–45	34 (58.6)	24 (41.4)	
	46–55	14 (50.0)	14 (50.0)	
	Above 56	7 (63.6)	4 (36.4)	
Gender	Female	33 (58.9)	23 (41.1)	0.204
	Male	74 (49.0)	77 (51.0)	
Farm location	Bova HA	28 (51.9)	26 (48.1)	0.162
	Buea road HA	15 (35.7)	27 (64.3)	
	Bokwango HA	6 (54.5)	5 (45.5)	
	Molyko HA	12 (63.2)	7 (36.8)	
	Muea HA	35 (62.5)	21 (37.5)	
	Buea town HA	7 (38.9)	11 (61.1)	
	Tole HA	4 (57.1)	3 (42.9)	
Level of education	No formal level	1 (50.0)	1 (50.0)	0.000
	Primary school	22 (88.0)	3 (12.0)	
	Secondary school	47 (57.3)	35 (42.7)	
	University	37 (37.8)	61 (62.2)	
Position in farm	Employee	8 (26.7)	22 (73.3)	0.012
	Business partner	4 (57.1)	3 (42.9)	
	Farm owner	95 (55.9)	75 (44.1)	
Farm size	Large	16 (36.4)	28 (63.6)	0.030
	Medium	30 (49.2)	31 (50.8)	
	Small	61 (59.8)	41 (40.2)	

HA=Health area

### Infection prevention and control practices among poultry farmers

Majority of the respondents (54.1%) had poor prevention and control practices on zoonotic poultry diseases. The poultry farmers attested using standard infection prevention practices including hand hygiene, change of clothes, foot baths and use of gloves, boots and masks in their farms. However, majority of the farmers did not use gloves (59.9%) and face masks (66.7%) (Table-4). Furthermore, most (54.6%) participants did not invite veterinary doctors or technicians to their farms.

**Table-4:** Participants’ zoonotic disease prevention and control practices.

Variable	Category	Proportion of farmers (%)
Veterinary doctor or technician visit and control	No	113 (54.6)
	Yes	94 (45.4)
Frequency of visit	Less frequent	13 (13.8)
	Frequent	62 (66.0)
	More frequent	19 (20.2)
Frequency of Hand wash	Not at all	34 (16.4)
	Sometimes	28 (13.5)
	Always	145 (70.0)
Use of gloves	Not at all	124 (59.9)
	Sometimes	49 (23.7)
	Always	34 (16.4)
Use of face mask	Not at all	138 (66.7)
	Sometimes	41 (19.8)
	Always	28 (13.5)
Wearing of boot	Not at all	64 (30.9)
	Sometimes	37 (17.9)
	Always	106 (51.2)
Use of footbath	Not at all	98 (47.3)
	Sometimes	29 (14.0)
	Always	80 (38.6)
Change of clothes	Not at all	75 (36.2)
	Sometimes	43 (20.8)
	Always	89 (43.0)

**Figure-5:**
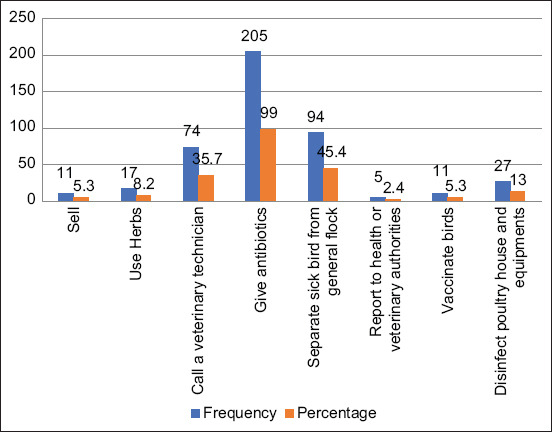
Common infection control actions taken by the poultry farmers on sick birds.

**Figure-6:**
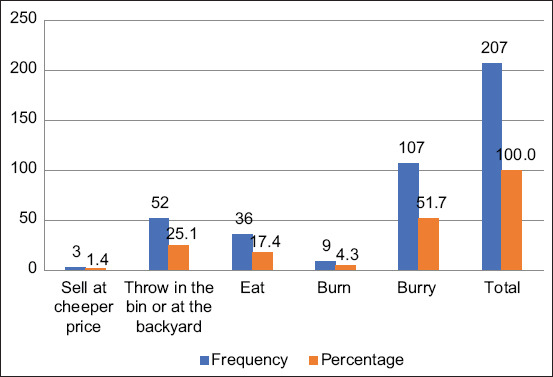
Infection control practices on dead birds.

Participants’ strategies to control the spread of diseases on the farms were evaluated by analyzing their reactions toward sick and dead birds and the results are recorded in Figures-5 and 6. The most common actions taken by participants with regard to sick birds were the use of antibiotics (99%) and quarantine of sick birds (45.4%); however, some participants would prefer to sell the sick birds (5.3%). Only few farmers would report to the veterinary health authorities (2.4%), vaccinated their birds (5.3%), or disinfected poultry houses and equipment (13%).

Participants were also asked how they disposed of dead birds (Figure-6). The most common response (51.4%) was “bury the bird carcasses.” However, some participants would sell the carcasses at cheaper prices (1.4%) or would eat them (17.4%).

The relationship between participants’ risk perception, knowledge, and prevention and control practices of ZDs was analyzed using Chi-square test and logistic regression as shown in Table-5. There was a significant association between risk perception and knowledge (p = 0.007; CI = 1.257–4.200) as well as between risk perception and prevention/control practice (p = 0.002; CI = 1.451–4.867). It was revealed that poultry farmers who had good knowledge of ZDs were about twice more likely to have high-risk perception than those with poor knowledge. Furthermore, farmers with high-risk perception were about 2 times more likely to exhibit good farm practices than those with low-risk perception.

**Table-5:** Association between participants’ risk perception, knowledge, and prevention and control practices on zoonotic diseases.

Variable	Category	Risk Perception (%)			p-value	AOR	95% CI	
	
		Low	High	Total			Lower	Upper
Knowledge	Poor	78 (69)	35 (31)	113	0.007	1.000	1.257	4.200
	Good	40 (42.5)	54 (57.5)	94		2.297		
	Total	118	89	207		NA		
Prevention and control practice	Poor	82 (69.5)	36 (30.5)	118	0.002	1.000	1.451	4.867
	Good	36 (40)	53 (60)	89		2.658		
	Total	118	89	207		NA		

CI=Confidence interval, AOR=Adjusted odds ratio, NA=Not applicable

## Discussion

The present study was initiated to determine ZDs knowledge, risk perception, prevention, and control practices among poultry farmers in the BHD, Cameroon using a one health perspective. There are increasing interactions between humans, livestock, and the environment [24]. These interactions often lead to increased risk of ZDs transmission from animals to humans [4]. Furthermore, as most of the small-scale farmers (in low-income countries) live below the poverty limits, they may have little or no education or skills in proper farm management. This might result to inappropriate knowledge, unawareness of raising livestock in a healthy manner and higher exposure to some potential risk factors associated with ZDs. Inadequate knowledge and low perception of the risk associated with these diseases may enhance poor prevention and control practices on the farms. This study made use of the “One Health” approach to include in the study poultry farmers and to sensitize various stakeholders of animal/human health in aspects of ZDs occurrence and risks in poultry farms, with the ultimate goal to provide insight to the proper management of ZDs in the study area.

The present study revealed that most poultry farmers in the study area were males, within the youthful ages of 26 and 35 years and were university graduates. Since agriculture forms the backbone of most developing economies, it is common to find persons of working ages engaging in agriculture for income generation and sustenance [5, 6, 25]. In addition, majority of the farmers in this study were farm owners who also served as farm workers but with little or no experience. Lack of financial resources could have accounted for the inability of the farm owners to hire trained personnel. Furthermore, the most common birds kept by poultry farmers in the study area were broilers which are usually meant for meat production. It was also observed that most of the farms in the study area were small in size with average flock capacity of about 500 and average yearly production of about 3000 birds. To meet the Sustainable Development Goal to eradicate extreme poverty and hunger [26], there is need for more poultry farms in our setup to increase their capacities so as to meet the increasing demand for poultry meat. Furthermore, the practice of mixed farming (with predominant animals being pigs) may enhance the development of antibiotic resistant strains [27] and this should be discouraged.

In this study, most (54.6%) of the farmers were not knowledgeable about ZDs and the findings are in agreement with some previous reports [22, 28]. The level of knowledge of ZDs and adequate infection prevention and control practices in farms are crucial in curbing the spread of infectious diseases among high risk groups. Awareness of farm workers on possible transmission of pathogens from animals to humans is essential in adopting adequate prevention measures. Although majority of the study participants knew that infections could be gotten from sick birds most of them did not know that infections could be acquired from apparently healthy birds. It is a fact that some microbes that occur as normal flora in birds and animals may become pathogenic in humans [7]. Consequently, the absolute unawareness of farm workers on potential infection transmission routes may present with high risk of exposure to ZDs in poultry farms.

Poultry ZDs commonly reported by farmers were salmonellosis, Newcastle disease, avian influenza, avian tuberculosis, and colibacillosis; and the predominant signs and symptoms included abnormal droppings, general inactivity, cough, and loss of appetite. Our results corroborate previous findings in which salmonellosis, avian influenza, Newcastle disease, and campylobacteriosis were commonly reported in poultry farms [28, 29]. Contrarily, our poultry farmers were seemingly unaware of the zoonotic potential of foodborne pathogens such as *Campylobacter* spp. Furthermore, poultry farmers in the study area had more than one source where they could obtain information concerning poultry diseases and ZDs; the most common being from other poultry farmers and media such as television, radio, and internet. These sources of information tend to be common for most aspects of daily life. However, information from these sources may be limited and non-specific. Sometimes, livestock farmers may need customized information related to their specific geographical settings. Such information can most likely be gotten from their local veterinary professionals or animal health workers; but this was not the case in our study. Thus, effective communication between farmers and veterinary professionals in the study area may help to improve risk perception and farm management practices. Evidence have shown that building partnerships with community-based stakeholders, providing trusted sources of information, and proper training of the target population are effective ways of ensuring health promotion activities in farming and rural communities [30]. The previous studies indicated that health education interventions significantly improved the knowledge of farmers toward control of ZDs [31].

Risk perception of ZDs was assessed by investigating participants’ feelings when exposed to potentially hazardous materials and their consciousness of getting infected on the farms. More than half of the farmers had low-risk perception of ZDs. Most of the participants considered coming in contact with dead, sick birds or bird’s faces as a possible source of ZD exposure or transmission. These results are similar to previous findings [21, 32] carried out among veterinary professionals. Nonetheless, majority of our participants did not consider contact with bird’s fluid (blood) or live birds as potential risk of infection. Such category of farmers are at high risk of acquiring and/or spreading ZDs since they will not see the need of wearing PPEs when handling live birds or during slaughter. Similar findings have been reported and this was attributed to poor knowledge of poultry diseases and potential sources of ZDs transmission in poultry [28].

The concern of the poultry farmers about them or their colleagues, clients, or family members contracting a ZD may be a reflection on their awareness and respect of biosecurity guidelines. A considerable proportion of farmers in the present study was concerned; however, this proportion was lower than that reported by Dowd *et al*. [33] among veterinarians. Veterinarians are more likely to be educated or trained on aspects of ZD exposure, prevention, and control compared to ordinary farmers. It was also noticed that more farm employees had high-risk perception than farm owners and business partners while those with university education had high-risk perception than participants with the lower level of education. Meanwhile, more participants with small farms (<500 birds) had low-risk perception compared with their counterparts with large farms (>1000).

Poultry farmers were interviewed on the preventive/control measures they use against ZDs in their various farms. Majority of participants had poor prevention and control practice level. This could be explained by the fact that most of the respondents were not aware of a possible transmission of a zoonotic infection from the live birds and other potentially contaminated poultry materials. A good proportion of the participants indicated they always washed their hands before and after contact with birds or poultry site and this is commensurate to previous findings [28, 32]. The importance of hand hygiene in reducing infectious disease risks among the farming community could be increased by encouraging farmers to wash their hands properly and at the correct time [30, 34]. However, a large proportion of farmers in our setup did not make use of basic prevention practices such as use of gloves, facemask, and/or foot-bath. This could be explained by their unawareness of possible airborne or other means of transmission of diseases from birds to humans.

Although most farmers would treat their sick birds with antibiotics and/or separate (quarantine) them from healthy birds, some would prefer to sell the sick or dead birds at a cheaper rate for consumption. Eating or handling of bird carcasses is a practice that could propagate the spread of diseases from the farms to the community; and it should be discouraged. Moreover, the fact that most farmers would not employ the services of veterinary experts during an outbreak is a cause for concern. A more worrying concern is the use of antibiotics by almost all the farmers. Worthy of mention is the fact that antibiotic use especially in the veterinary sector in Cameroon is seldomly regulated. As a result, these antibiotics are readily availability at feed mills and veterinary stores at affordable prices. In such scenarios, antibiotics can easily be misused or overused and this might enhance the development and spread of antimicrobial resistance [35]. There is a need for a sentinel institution to be established in our setup, to regulate the use of antibiotics in farm animals as well as to set guidelines for proper farm practices in a bit to curb the spread of ZDs.

## Limitations of the study

This study had the following limitations:

- The snowballing technique used to enroll study participants might have triggered some bias in the study since the researchers depended mostly on referrals from already identified farmers.
- The scarcity of day-old chicks during the study period might have influenced the category of farmers enrolled into the study.

## Conclusion

Significant relationships were observed between participants’ risk perception, knowledge, and prevention and control practices of ZDs. Poultry farmers who had a good knowledge of ZDs were about twice more likely to have high-risk perception than those with poor knowledge. Furthermore, farmers with high-risk perception were about 2 times more likely to exhibit good farm practices than those with low-risk perception. From a “One Health” perspective, education and sensitization of all stakeholders in the study area through training workshops and constant follow-up of farmers by veterinary professionals will help boost farmers’ awareness of ZDs and improve their mitigation practices.

## Authors’ Contributions

MEAB: Conception and design of the study, data validation, revision of the manuscript, general supervision. JCNL: Conception and design of the study, data collection, data processing and analysis, drafting and revision of manuscript. EA: Data validation, revision of the manuscript, general supervision. RBN: Data collection, data processing and analysis, drafting and revision of manuscript. NT: Data analyses, data validation, general supervision. All authors have read and approved the final manuscript.
